# The Hospital. Nursing Section

**Published:** 1904-09-10

**Authors:** 


### The Hospital.

**Nursing Section.**

Contributions for this Section of "The Hospital" should be addressed to the Editor, "The Hospital," Nursing Section, 28 & 29 Southampton Street, Strand, London, W.C.

No. 937.—Vol. XXXVI. *SATURDAY, SEPTEMBER* 10, 1904.

### Notes on News from the Nursing World.

### QUEEN ALEXANDRA'S MILITARY NURSING SERVICE.

We are informed that the following staff nurses belonging to Queen Alexandra's Imperial Military Nursing Service have been appointed sisters:—Nurses M. M. Bond, A. F. Byers, E. M. Denne, M. Kendall, S. B. Lanyon, E. M. Pettie, C. G. Stronach, and A. A. Wilson. Miss E. H. Ho[illegible]dley, sister, and Misses E. Barber, S. K. Bills, B. N. Daker, and G. Knowles, staff nurses, have been confirmed in their appointments; and Misses F. A. Dawson, E. M. Perkins, G. M. Smith, and A. M. MacCormac have been provisionally appointed staff nurses.

### RUSSIAN NURSES AND RAILWAY TRAVELLING.

The nursing sisters who have been sent from St. Petersburg to Hai-cheng suffered so much in the shape of exasperating delay in their long railway journey, lasting upwards of six weeks, not to speak of a plague of flies, that, on their arrival at their destination they confessed that they looked forward to the mere hardships and deprivations of the field as to comparative luxury. It was a common thing for the train in which they travelled to be shunted into a siding and stuck there for half a day.

### FOR THE HONOUR OF THE UNIFORM.

If a nurse wears her uniform out of doors she should be scrupulously careful not to bring even the shadow of discredit upon it. The superintendent of a well-known nursing organisation, whose letter appears in another column, shows that there are nurses who do not take this view. We venture to suggest that she should make very strong endeavours to improve the general "tone" amongst her nurses, so as to effectually make it impossible that any reproach should be cast upon them. We feel sure that if the matron has the confidence of her staff, she will speedily embue them with a wholesome *esprit de corps,* which will make them as jealous as she is herself of the reputation of the hospital.

### THE MATRON OF UITENHAGE HOSPITAL, CAPE COLONY.

We understand that Miss Edith M. Brawn has been appointed matron of the hospital at Uitenhage, Cape Colony. The hospital is a quite new institution and contains forty beds. Miss Brawn, who has been in South Africa for some time, was trained at the Walsall and District Hospital. She was then successively staff nurse, out-patient nurse, and night sister, at the Branch Seamen's Hospital, Royal Albert Docks, London. For the last two years she has been theatre sister at the Somerset Hospital, Capetown, and she holds a teacher's certificate of cookery and housekeeping from the Birmingham and Midland School of Cookery.

### BRIGHTON WORKING MEN AND THE QUEEN'S NURSES.

The annual parade of the Brighton Friendly and Trade Societies in aid of the local medical charities and the Queen's Jubilee Nursing Association was remarkably successful, the amount collected being £178 8s. 8d., or £63 5s. 11d. more than last year. Most of the amount was contributed in copper, and a notable feature was the readiness with which the working men responded to the appeal made to them on their way to dinner. The nursing association is badly in need of financial assistance, and the substantial help rendered by the class who derive the most benefit from it is very encouraging to all the workers.

### PRESENTATION TO MAJOR BALLANTINE.

Prior to the departure of Major Ballantine from Manchester Workhouse and Poor-law service on a pension, he was the recipient of several marks of esteem. The superintendent of the nurses and the nursing staff gave him a drawing-room chair, beautifully upholstered in tapestry; and other articles presented were a handsome gilt timepiece by friends connected with St. Thomas's, Lower Crumpsall, and a suite of dining-room furniture by the medical staff, the officers of the house, and various officials connected with the institution. The presentations were made on separate occasions, and speeches of a very cordial character were delivered. The steward of Crumpsall Infirmary spoke of the high esteem in which Major and Mrs. Ballantine were held by all; and Dr. Tylecote, on behalf of the medical and nursing staff, testified to the advice and assistance they had received from the retiring master, who, in well-chosen language, acknowledged the various gifts.

### A NURSE SAVES THE LIFE OF A CHILD.

An interesting incident at the last meeting of the Whitechapel Board of Guardians was that they directed that a letter be sent to Miss Woods, a nurse who is employed at the schools of the Board at Grays, Essex, thanking her for her devotion in the case of a sick child, with whom she had sat up for four days and four nights. In fact, the doctor reported that the life of the child had been saved by her unremitting attention. No doubt the true reward of the devotion of Nurse Woods is the recovery of the child, but it is pleasing to record the fact that it has been officially recognised.

### GOOD FAITH BETWEEN MATRONS.

There cannot be any difference of opinion as to the absolute necessity for good faith between matrons and superintendents of hospitals and nursing homes. Unfortunately, however, it is not always strictly observed. Not long ago we mentioned a regrettable instance of breach of good faith, and we hear now of another. The matron of a home in the South of England applied to another matron on behalf of a nurse who wished to join her staff. She asked the usual questions about sobriety and propriety, and received a favourable answer. On intimating to the nurse her willingness to engage her, the nurse quite civilly refused the appointment. The matron naturally pressed her for her reasons, and she then stated that she had heard that the matron had made inquiries as to her sobriety and discretion, and that she took exception to such action. We think that the nurse was very easily offended, but, of course, the matron to whom the inquiries were addressed should not have mentioned their character to the person whom they concerned.

### THE PRIVATE NURSE AND HER DUTIES.

We do not think that the views which have prompted Miss Besley's letter of protest under the above heading will be shared by many of our readers. It may be true that Miss Besley has no need of the advice given, but it is certain that there are other nurses who may benefit by the warning given by "A Physician." It is true that all human beings when ill are entitled to every consideration; it is equally true that they do not always receive it "in hospital" owing to pressure of work on the nurses. In private nursing, when there is only one patient to attend to, the nurse must give him more attention than she would give to a hospital patient, and it is this fact which the nurse in her first experiences of private nursing is apt to overlook. As it is the physician who supervises the nurse's work, he is the very best person to give advice on the subject of private nursing.

### THE NURSES OF KINGSTON UNION INFIRMARY.

A very satisfactory report has been issued by Mr. James Cantlie in reference to the examination held by him of the probationers at Kingston Union Infirmary on August 30th. Certificates of proficiency in nursing have been awarded to Nurses J. Wilson, E. Barber, E. N. Watson, E. W. Barter, C. M. Simkins, R. Harman, Jackson, and M. Smith. Each of these nurses, except Miss Harman, also holds the certificate of the London Obstetrical Society. Mr. Cantlie states that "the nurses have been well taught, theoretically and practically," that their knowledge of anatomy and physiology is "extensive and accurate," and that their knowledge of practical nursing and ward hygiene is "excellent." His report reflects credit not only upon the probationers, but also upon Miss J. A. Smith, the matron of Kingston Infirmary.

### NURSING ARRANGEMENTS AT TAUNTON INFIRMARY.

The Local Government Board very properly declined to sanction the appointment of a nurse at Taunton Workhouse Infirmary on account of her youth and inexperience. By the steady pursuance of this policy the board will do much to raise the standard of nursing in the small Poor-law infirmaries. No doubt they acted upon the advice of their inspector, Mr. Preston Thomas, who lately inspected the workhouse at Taunton and found that only a head nurse and a second nurse were employed. Mr. Thomas has since suggested that the head nurse, who has had a good hospital training, should be appointed superintendent nurse and have a couple of properly trained nurses under her, and this suggestion has met with the approval of the Taunton Guardians.

### OUTING FOR LEICESTER NURSES.

By invitation of Mr. A. P. More, a number of the nurses attached to the Leicester Institution of Trained Nurses had an enjoyable outing at Benscliffe the other day. Accompanied by the Lady Superintendent, Miss J. M. McHardy, the party drove in brakes to Benscliffe, and on their arrival were entertained by Mr. More. The afternoon was passed in the gardens and later on the nurses went through the picturesque grounds of The Brand. Altogether the proceedings were of a most enjoyable character.

### JARROW NURSING ASSOCIATION.

In the fourteenth annual report of the Jarrow Nursing Association, which has just been issued, it is stated that during the past year 2,139 visits were made to poor patients. The financial position is very satisfactory, the total income from all sources being £89 18s. 1d., and the balance in hand £30 0s. 7d. A feature of the period was the receipt of £10, the proceeds of a ball which was held in the town on behalf of the organisation.

### COLLECTORS WANTED AT BURGESS HILL.

The importance of an adequate number of collectors is shown in a striking manner in the thirteenth annual report of the Burgess Hill Nursing Association. A considerable falling off in the amount subscribed by the cottagers is announced, and this is attributed partly to the fact that there were several districts without collectors. The balance in hand has thus been reduced from £73 9s. 9d. to £54 19s. 5d., but the work done by the Queen's nurse is so excellent and is so much appreciated that, given a satisfactory response to the appeal for more collectors, we have no doubt the Association will soon regain the ground it has lost.

### The Nursing Outlook.

"From magnanimity, all fear above;
From nobler recompense, above applause,
Which owes to man's short outlook all its charm."

### HEALTH LECTURES IN PRISONS.

The pleasant news that Miss Rossie, health lecturer to the Hants County Council, has been allowed to give a course of lectures to the women in Portsmouth Gaol, should stir nurse-lecturers in other counties to take up this good work. But we must enter a strong protest against two phrases used by Miss Rossie; she says in comparing the women at Portsmouth with the women who were lectured to at Wormwood Scrubs that the latter are "a more essentially evil type; they are so soaked in criminal associations that it is hopeless work to try to show them the dignity of home-keeping"; and again—"wholly irreclaimable, like those at Wormwood Scrubs."

Now there appeared in these pages at the time the lectures were being given by a nurse at Wormwood Scrubs, an article from the pen of Mrs. Hawksley, wife of the vicar of All Saints', Portsmouth, who had attended one of the lectures, and Mrs. Hawksley insisted very strongly on the good done by the lectures and on the good behaviour of the prisoners. Indeed, everyone who saw how eager the Scrubs women were to learn, how valiantly they tried to reverse a bandage and tie a reef-knot, was struck by the fact that these prisoners had evidently many elements of good and were far from being irreclaimable or soaked in crime. If Miss Rossie needs further evidence we can refer her to the articles in the *Graphic* and *Westminster Gazette*, or, better still, here is the hitherto unpublished testimony of the Governor of Wormwood Scrubs, given in a letter to the nurse:—"Now that your course of health lectures is concluded, I must express to you my thanks, and also that of the officers, for the trouble you have taken and at the same time assure you that (from course of conversation with a few prisoners) the women have derived much benefit from the lectures."

It is necessary to insist on taking the hopeful view of the sick in soul, as much as those of the sick in body; the element of hope is essential for all successful work, and the prison lecturer must take sympathy and compassion with her if she would meet with the success she deserves. As a rule it is the fault of the officials and not of the prisoners that causes difficulty with regard to improvements. It will be within the memory of most of our readers how, only a year ago, some 200 nurses volunteered for prison work as warders, and not one was accepted—though it is impossible to believe that all those 200 were unfitted. The women prisoners have now been removed from Wormwood Scrubs and are all crowded at Holloway—some 800 of them are ever there. And the scheme of lectures by the lady visitors has already broken down, and their place is taken by people of the district-visitor type, who are sent by the visiting chaplain. About once a fortnight some 40 out of the 800 women, have a goody-goody talk given to them in this way, and this is what the Prison Commissioners insist on calling a lecture scheme. And yet trained and experienced teachers have been offered them, and the late London School Board offered to run an evening school on strictly official lines.

But no! there behind the walls are those 800 women, some 70 per cent. of whom when released will return to prison again, because their treatment is not reformatory; and here on this side are nurses and teachers full of sympathy with the sick of soul, longing to give a helping hand to their sisters who have stumbled by the way; but between the two classes the prison official has reared a fence of red tape. It is cruel and unjust, but so long as all Prison Commissioners and inspectors are men, it seems hopeless to look for sympathy with women.

Only last month Mr. Claude Hay asked a question in the House of Commons about two women who during detention before trial at Holloway had had their hair cut short because it was dirty. The solemn reply of the Home Secretary was that this was necessary, whereas any nurse could have told him how to clean a woman's hair without subjecting her to having it cropped.

There is no better way of rousing kindly instincts in the woman prisoner than to teach her how to tend a sick child or a bed-ridden old man—put it in her power to do a kind action, and enthuse her with stories of kind actions, and you have probably started her on the right path. And then teach her simple cookery and sewing, and when she goes home she may possibly try to keep her home more comfortable; or, if the poor wretch has no home to go to, it will be easier for her to get work and earn an honest living. May nurse-lecturers in all the big towns at once write to the gaols and ask permission to try to teach hygiene and humanity, health of body and soul, to the women in prison. For our part, if only the Prison Commissioners will allow it, we are ready at any moment to supply to Holloway, free of all charge, trained teachers not only of hygiene, cookery, and needlework, but also of all other simple subjects. But the subjects must be simple and simply taught, and appeal by diagrams and experiments must be made to the hand and eye. Mere lectures are not enough; let the voluntary lecturer be sure she can teach and have plenty of appliances, for though her audience are not irreclaimable they are probably very ignorant.

### Lectures Upon the Nursing of Mental Diseases.

By Robert Jones, M.D.Lond., B.S., F.R.C.S.Eng., M.R.C.P.Lond., Resident Physician and Superintendent of the London County Asylum, Claybury.

### LECTURE XVIII.

The private nurse in a private house is face to face with risks and difficulties which nurses in institutions never meet. The chief of these are less from the nature of the case than from the greater difficulty of controlling a patient in her own house where she has been mistress, and where she is more apt to resent interference with her liberty. Difficulties will also arise from the probable interference through nervousness, suspicion, and fear on the part of the relatives and friends, and a nurse will have to exercise considerable tact and forbearance, and maintain a calmness under much provocation whilst nursing private cases. It is well for the medical officer to issue his instructions in writing, and to protect the nurse he should take all responsibility for the instructions being carried out. It is still better for the medical officer to use his influence to keep the patient and her relatives apart, as their presence will tend greatly to prolong the illness and to retard recovery. If the patient exhibits violence the nurse should as far as possible try to prevent, in a private house, every kind of impropriety of manner and language, and should, if necessary, summon aid as quickly as possible; in the meantime she should avoid personal struggles, and endeavour to tranquillise the patient by kind words or by diverting her attention. It is often better to persuade such a patient if possible to enter her room, where the quietude and the unexciting surroundings may restore her calmness.

A private nurse takes much pains as a rule to acquire a knowledge of the character of her patient, whom she encourages to good conduct and to habits of neatness and order, securing her confidence by friendly treatment and a uniform regard to her comfort. A nurse should always heed every complaint made by a patient, however trivial it may appear. It is important in a private house to promote such habits as may prevent patients being negligent of cleanliness when in bed, and this can only be done by proper attention paid during the night.

For changing the under bed-linen of patients who are kept in bed there are two methods usually practised by nurses—viz., either (*a*) replacing the sheet and blanket lengthwise, or, when patients cannot be turned on their side (*b*) across the bed from the foot upwards. For the first-named method the pillow is removed, the patient rolled on one side beyond the middle of the bed, the soiled sheet and blanket are rolled their whole length until the roll is placed right against the patient's back, the clean sheet and blanket are then half-rolled along their whole length, the two rolls of clean and soiled being thus side by side. The free unrolled portion of the clean sheet and blanket are then placed in the proper position over the bed, mattress, or mackintosh, and tucked in under. The patient is then rolled back to the clean linen, the body passing over the two rolls (clean and soiled) which are now free. The soiled bed-clothes are further rolled outwards and removed and the unsoiled sheet and blanket follows, the free edge being tucked in on this same side. If the patient cannot for any reason, such as fracture of the leg or thigh, etc., be moved from one side to the other, a similar process of changing takes place across the bed from the foot towards the head.

To remove the upper sheet without uncovering the patient the bed-clothes are stripped except the sheet and blanket. Over these the clean sheet is placed, the soiled one is drawn out underneath, and afterwards the blanket, so that the clean sheet is now next to the patient. The remainder of the bed covering is placed over this in the usual order, and the changing is thus completed.

The draw-sheet, used for helpless patients to protect the lower bedclothes, is a small sheet folded so as to reach from the middle of the patient's back to the knees, the sides being well tucked in under the mattress. When soiled the draw-sheet is removed in the manner indicated for the lower sheet, or if the patient is not too heavy, she can be lifted either by the nurse alone, who passes her arms well underneath, one under the back the other under the knees, whilst another nurse places the draw-sheet in position, or two nurses can do this by joining hands on each side of the bed. It is necessary to see that all bed-linen is well aired before being used. In using the bed-pan—the slipper for women and the round pan for men—a flannel covering for the sides is always adopted to prevent the cold earthenware coming into contact with the patient's body. Carbolic solution, Jeyes' fluid, or some other disinfectant is always placed in the pan after use. This should also be done after washing the pan, and the nurse must see that none is left on the sides of the pan to irritate the skin. It is usual to keep the excreta in the lavatory for inspection by the medical officer.

No harsh treatment nor any punishment may at any time be used to a mental patient, nor should any violent or intemperate language be used to a patient. When certified, such persons are carefully guarded by the provisions of an Act of Parliament and the nurse or person who ill-uses the insane is liable to severe penalties. The whole time of a private nurse is devoted to the patient, and she pays attention to the patient's food, dress, occupation, exercise, amusement, and general conduct. Another difficulty which the private nurse has to contend against results from the structure and the arrangements of the private house in which the patient resides, and especial care must be taken to guard against risks from stairs, open windows, razors and knives, medicines, poisons, etc. These latter have to be carefully put away, and the nurse must also be cautious to see that ladders, steps, or other objects with which patients may hurt themselves or others, or by means of which they may escape, are placed out of the reach of their patient. The private nurse is always expected—more so even than the public nurse—to set an example of order, quietness, kindness, and good conduct to the patient, and to be careful of what she says concerning the house, relatives, or others in attendance. She should herself set an example of usefully employing her time, as her conduct will probably become the standard by which the patient orders her own acts and conduct. Like the public nurse, the private nurse will be expected to pay strict obedience to all orders from the medical officer in charge, and it is undesirable that she should talk of the behaviour of the patient under her care; neither should she convey any letters or newspapers from her patient to any other person without permission from the medical officer in charge, or from the relatives or other persons appointed—in some cases by the Court of Chancery—to supervise the patient's care. It is very necessary for the private nurse to report all daily changes in her patient. To do this means much practice and close observation, but when carried out with precision and without exaggeration it is a good proof of valuable training. For this purpose she will keep a private daily memorandum-book recording (*a*) the food and drink taken, and the state of the bowels; (*b*) the amount of exercise and length of time in the open air; (*c*) the patient's temperature, pulse, and respiration (*d*) the weight and variations thereof

### LECTURES UPON THE NURSING OF MENTAL DISEASES—*Continued.*

observed from time to time; (*e*) the amount of sleep; (*f*) the chief mental symptoms, and whether any impulses hurtful to herself or others have developed; (*g*) any struggles, accidents or casualties that may have occurred; and (*h*) the administration of medicines.

In the administration of medicines a measure-glass, marked in teaspoonfuls, or tablespoonfuls, is used. This measure-glass holds two ounces as a rule, viz., the quantity contained in an ordinary wine-glass. A teaspoonful is the same measure as a drachm, two drachms are a dessert-spoonful, and a tablespoonful is half an ounce. A minim is one measured drop, and there are 60 drops to the teaspoonful or drachm. It is not often that a nurse is called upon to measure minims or drops when she is provided with a minim measure, but she must be careful in measuring out medicines of all kinds, as grave and fatal errors have occurred from the want of due care in this respect. Before pouring out medicines she should always carefully read the instructions on the bottle and be certain she understands them, and note the time they are to be given and shake the bottle before pouring. In pouring out medicines she should hold the bottle with the label uppermost, so as not to soil or obscure the instructions on the label by the medicine running over it after or whilst it is tilted. Medicines and external applications must be kept separate, and the medicine-glass carefully washed after using. If the medicine is nauseous a biscuit or crust of bread or peppermint will remove the taste.

It is well to know the actual quantity of nourishment taken, and for this the capacity of different holders should be known; for instance, a small teacup holds about four ounces, and a tumbler about half a pint. When insane patients are unable to sit up in bed they are fed either with a spoon or by means of a feeding-cup. The feeding-cup is half covered, it has a handle and a spout; the latter is long and curved, more especially when used for those who cannot lift their heads from the pillow. Too much fluid must not be put into the feeding-cup at one time, as it may run over the side and soil the personal clothing or the bed-linen. Feeding insane patients is one of the most common forms of duties the mental nurse is called upon to do. For mental cases in private houses the meals should be cleanly, nicely, and punctually served, more especially as many patients entertain delusions of suspicion and poisoning in regard to their food, or they refuse food from depressing delusions of personal unworthiness and wishing to die. In such states all the persuasive powers of tactful persons may be of no avail. Much, however, may be done by encouraging and coaxing the patient, who, when once she has tasted the food, may overcome any prejudice in regard to it and take it freely. The nurse has to be especially careful in feeding helpless or paralysed patients, who should be gently raised by passing the left arm behind the pillow or the shoulders, raising the pillow and head together, giving the fluid food with the other by means of the feeding-cup or spoon, being watchful that no liquid trickles into the lungs to set up broncho-pneumonia. In all feeding cases a napkin or towel should be used to protect the personal and bed-linen, as already stated, and the mouth should be wiped after each feeding. In cases of profound exhaustion it may be necessary to administer nourishment at frequent intervals, as the life of the patient may depend upon this; such nourishment as beef-tea, mutton-broth, albumen-water, egg-flip, milk, or stimulants may have to be given every two or three hours, and by a judicious varying of these the nurse may succeed in not nauseating the patient. It is surprising what quantity of concentrated liquid nourishment can be taken in the extreme exhaustion of mental cases, and it is equally surprising how apparently hopeless cases rally, obtain sleep, and mend under this treatment. When the mouth and lips are parched, and the teeth black with *sordes*, glycerine and borax or lemon juice and water can be used to cleanse them. In cases of melancholia, liquid nourishment administered in the early hours of the morning is the best antidote to depression, and if the habit can be broken the patient will probably begin to mend. As a rule, except when there is acute gastric disorder, water can be given to patients whenever they ask for it. Forced feeding with the nasal or œsophageal tube must never be resorted to by the nurse, whether public or private, but she is expected to get everything ready for the medical attendant to do this, viz. a glass or other funnel, a long rubber tube, carbolised vaseline, basin, soap and water, and the nourishment and medicines as ordered. Artificial feeding by the rectum may have to be performed by the nurse when "enules" are prescribed, or a small injection (about 4 oz) of peptonised beef-tea or milk is ordered every four hours. The private nurse may be confronted with many emergencies, certainly more than fall to the lot of public nurses, and find that she is expected to cope with them single-handed. Some of these are through attempts at suicide by hanging, drowning, burning, scalding, poisoning or suffocating, and there may be accidents of various kinds needing a resolute nerve. It is only after a full and complete training that the knowledge and experience necessary for coping with emergencies can be obtained, and the best training for a private nurse is obtained in institutions where all varieties of insanity and all kinds of cases are received and treated, and where these emergencies are experienced. In regard to one point, the question of stimulants, it is much the best plan for private nurses to abstain from these during the period of their engagement, as their conduct may be liable to misinterpretation. If the nurse be true to herself it follows, "as the night the day," that she must be true to her charge.

### Nursing in an Irish Workhouse Infirmary.

BY A COSMOPOLITAN NURSE.

Upon arriving at the infirmary my first introduction was to an old lady with a dislocated arm; evidently she was a stranger to soap and water. While an "attendant" was getting me the requisites for washing my old "Biddy," I tried to make friends with her, by inquiring about the accident, and giving sympathy, the best of all medicines. When all was ready I ventured to say in a persuasive tone, "Now you will let me wash you, won't you?" "Faith an' I won't," was the answer; "I was always saller, an' all the washin' in the worrld won't make me white." I explained that it was cleanliness, not whiteress, we wanted; but she remained obdurate. "I wash my face on a Sunday, an' that's enough, shurre." I coaxed, and tried to induce her to feel the comfort of it, in vain. "I tell ye, ma'am, I was born saller, an' ye can't wash me white," she said decisively. "But," I argued, "I washed the black people in India, and you know I couldn't get them white." "Musha now," said Biddy, conquered at last, "an' did ye come from furrin' parts to wash me?" "And why not?" was my response, and while talking on the ways and customs of "them black people in furrin' parts" I had my old lady successfully washed, and more, received her grateful thanks. However, the triumph came later when she asked to be washed, and whispered to me, "Wouldn't I go to th' other ould woman an' wash her, for mebbe she'd be jealous." From that time to this, Biddy and I have been fast friends, and when I took her down some pictures and photographs of "furrin' parts," she was highly elated. "Musha Alanna, an' how could ye leave that beautiful land, to come to this hungry land?" "To wash you, of course," I answered laughing, and Biddy turned to a friend and said in a loud proud whisper, "She come from furrin' parts to wash me, an' shure the sky an' the say are the one pattren there," she added, pointing to a coloured picture of coast scenery.

Old Mary and her Tobacco.

My next charge was old Mary, who hailed me as I was passing down the ward, "Whist, come here till I tell you, let me whishper it in your ear," and she seized my two hands, and drew me towards her, and then in an audible whisper, "I'm a poor, ould woman, without a pinny in the worrld, and I'm axin' ye, because I know ye'll give it." Then in a real whisper, "Won't ye give me a bit o' baccy?" The appeal was irresistible, and I promised I would get her some the next day. "Shure, I knew ye would," she said, satisfied at her own discrimination. I took it to her the next day, a whole half-ounce of baccy, which was to her a large supply, and cautioned her not to smoke in bed, though I was told that it was permitted in this infirmary. Her gratitude was unbounded, and all the blessings of Heaven were called down upon me, and a promise to pray for me "on her bended knees" every night. The Sister who was with me asked if she was not to have any prayers. "Ah, shure," said o'd Mary, "I always pray for the poor sinners"; upon which I told sister she might take a back seat if she had no "baccy" to give. Old Mary says, "I'm am ould, ould woman." "How old are you, Mary?" I asked one day. "Sure, an' how would I know?" she said, "but me mither tould me that when I was a year an' a half ould, that it was the Irish Rebellion, an' she took me up in her arms an' ran out with me into a great brake of briers, an' what leapt out over us but two soldiers, and they went on widout seeing us, an' burnt and kilt all before them, an' how ould does that make me, ma'am?" "That makes you 107½ years old, Mary," I told her. She looked at me startled, and then clasping her hands and looking up to Heaven, said, "Oh, Merciful Jesus (reverently bending her head and knees), have pity on me poor ould sowl and body; I didn't know I was so ould!" Every Sunday I have to give old Mary her tobacco. "I can't live widout it, alanna," she says, if I expostulate with her; and, indeed, it would be hard to break off the habit at 107½ years of age! She is able to be up every day, and goes down to the "yard" to smoke her pipe; her only other request is for "a tippeen o' tay, an' a grain o' sugar," "for I do be so cowld in mornin' early," she explains.

Gratitude of the Irish Poor.

It is surprising to see the quiet resignation of the Irish poor; they accept their sufferings, hardly expecting any relief; their gratitude for every little service is great. If you straighten the bed, the usual method of thanks is, "Musha, may the Lord give ye a bed in Hiven;" if you open a door it is, "May the gate of Hiven be opened to ye!" and so on for every little service rendered. One woman was brought in in a very unclean state, covered with vermin. She was a middle-aged woman, and I tried to make her realise the shame of so neglecting herself, but was only met with "Sure an' isn't it a consolation to have them now, rather than when ye are 'wakin' (dead)," she said, looking up with an expression of having given a conclusive answer. The bath, and anything to do with soap and water, is a continual battle. "Sure I'm not a lunatic," said one, "an' why should I be bath-ed?" (they never say bathed in Ireland), and, though they submit to hospital regulations, they go back to their unwashed state as soon as they go out.

### Ward D 3.

The Ward Sister leans over the balcony and gazes wistfully eastward where the sky glows dimly red as it reflects the glare of the lighted shops. For a moment she has forgotten the long double row of beds in the ward behind her, and breathes the cold night air with delight; but her brief spell of rest, physical and mental, is all too short. A fretful insistent wail comes to her ears, and she hastily opens the long glass doors, lifts the curtains that conceal it, and re-enters the infirmary.

"Sister, don't yer hear Amelia a-calling yer?" says a husky voice from the bed nearest the door, a post of honour occupied by the oldest inhabitant, an aged dame of 90, who has been an inmate of the ward for 10 years.

The Ward Sister nods cheerfully, for Mrs. Methuselah, as the nurses call the oldest inhabitant among themselves, has to be humoured a good deal. She knows what is due to the widow of a man who paid rates and taxes regularly for 40 years, and has helped to wear out more than one nurse since her instalment in the infirmary. Amelia, who is fretfully wailing for the Sister, is a child compared with Mrs. Methuselah, being barely 80. Just now she wears a fretful expression, and the corners of her mouth are turned down as far as they will go.

"Well, Amelia, what is the matter, now?" asks the Sister briskly.

"I want a sweet," says Amelia, plaintively.

"Well, you cannot have one," is the retort, and the Sister turns away, only to be recalled by a whimper.

"I do want a sweet," says Amelia.

"Now Amelia," the Sister descends to argument, "You know you had a sweet last night and were sick afterwards."

Amelia's blue eyes, still wonderfully and beautifully blue, fill with tears. "I won't be sick to-night," she promises.

The Sister wipes the eyes of her charge tenderly and continued: "You had a nice cup of tea only a little while ago."

Amelia pouts, and her withered hands move restlessly up and down the coarse coverlet. "Tea is afternoons, sweets is evenings," she declares querulously.

"You may have some grapes," says the Sister soothingly; but Amelia is not amenable to bribes, and reiterates her request for a sweet until a bright idea strikes the Sister and she offers a compromise—two grapes and half a peppermint. The old woman is graciously pleased to accept the offer, and beams upon her neighbours as she mumbles the hardly-earned lollipop.

"There; now you will be good and amiable, won't you," demands the Sister, and Amelia offers her a kiss and audibly blesses her.

Close by lies "Gran." She has only been in the infirmary a week, and the significant order of "Visitors at any time between 6 A.M. and 10 P.M." has been issued, for she is also in the eighties, and has a broken leg and a fractured thigh with which to contend. She has a visitor now, the only visitor she ever has, and a slight pucker disturbs the Sister's white brow as her eyes fall on the girl by the bedside.

"Madge" is one of those restless atoms of womanhood that great cities sometimes breed, finding compensation for hard work and little pay and a lack of pretty things, in books and plays; the fact that she works all day in a copying-office does not deter her from feeding her intellect wildly in her spare time, every moment of which is filled to excess. Even on Sundays she takes no rest, hearing some great preacher in the morning, attending some ethical lecture or Socialistic debate in the afternoon, and finding some other form of mental diversion in the evening. The Ward Sister wonders whether the girl is really capable of realising that Gran is on the outside edge of things.

"Isn't she wonderful? Why, she's quite herself to-night and has been telling me not to stay out late in quite her old style," says the girl eagerly as she catches the Sister's eye. "I tell her she will be up soon."

The Sister busies herself with Gran's medicine and makes no reply.

"And she isn't in any pain now, are you, pet?" rattles on the gay young voice.

"Except when we have to move her; then it's agony, not pain," puts in a short nurse who has come to the bedside and regards Madge with ill-concealed impatience.

"She is very brave and such a dear," the Sister added hastily, and the girl gives her a grateful glance as she bends over the bed to say good-night.

"I shall run in and see you in the morning, pet."

"Please God," murmurs Gran.

But Madge will have none of that, and mutters rebelliously that God has nothing to do with it as she follows the Sister down the ward. "She thinks I am selfish, that short nurse," says Madge, rather breathlessly, when the door is reached. "And I am, I know I am. I don't want Gran to die. She's all I have left and I don't *want* her to die."

The ward sister is dumb. As she returns to the ward she stops to speak to "Tommy." This is the second oldest inmate and has to be kept as far as possible from Mrs. Methuselah on account of the deadly jealousy that exists between them. "Tommy's" real name is Mrs. Atkins, but the younger nurse, a tall girl with a splendid face, will have a nickname for everyone. This particular nurse is just now sitting on the foot of Tommy's bed with "The Baby," a chubby infant of some six months old, to whom the nurse is far more devoted than is its mother, who is suffering from gastritis, and spends most of her time fretting for "My old Man." Next to Tommy is a sturdy urchin with a bandaged head who is engaged making a set of soldiers march to the tune of "Soldiers of the Queen," which he whistles in true street-arab style. As a matter of fact Dick Nobbs is well enough to get up, but he is so unmanageable that to keep him in bed seems to be the only solution to the problem of keeping him in the ward at all.

The tall nurse soon takes her departure to perform "The Baby's" going-to-bed ablutions, which she does in the middle of the ward in order to give everyone an opportunity of admiring the operation. This most of them do, with the exception of Mrs. Methuselah and Amelia, both of whom are jealous of "The Baby," who, happy mite, lies on the floor and kicks mightily while the nurse prepares her bath, gurgling with joy every time that young person sweeps by with a snap of her fingers and a shrill whistle. Probably "The Baby" will never have such a good time again nor be such a person of importance. Her progress back down the ward partakes of the nature of a triumphal procession; even the old woman whom the tall nurse dubs "Mrs. Gummage" smiles as the baby is deposited on her bed while the pillows of the red-curtained bassinette are shaken up and she shares in the last good-night romp before the mite is tucked up with the injunction to "go to sleep," a command which it has not the slightest intention of obeying and against which it protests loudly. Neither of the nurses can refrain from a caressing word or two as they pass and re-pass, preparing the patients for bed, and one is tempted to believe that the Sister's hands are aching to take it up and soothe it to sleep with some soft lullaby, but this final joy is denied the Ward Baby for discipline's sake, and the shrill protests drop to a sleepy murmur till at last it falls asleep so soundly that even the short, shrill screams of one or two of the women as they are prepared for bed fail to rouse it.

A little later the nurses foregather over a cup of tea as they wait to be relieved; sometimes a low moan breaks the silence, but Gran is silent, lying with patient eyes and weary face waiting for the dawn and praying that she may be spared yet another day to see her little Madge.

### Presentations.

On Thursday, the 1st instant, a pleasant ceremony took place at the Union Infirmary, Stockport, when the medical superintendent, Dr. Bale, on behalf of the nursing staff, presented the home sister, Miss G. Boylan, with a silver tea-and-coffee service, on the occasion of her leaving the above institution to be married. In his remarks Dr. Bale spoke most highly of the way in which Miss Boylan performed her duties, and said that the nursing staff and himself regretted her leaving, but wished her every good wish for a happy future.

### TRAVEL NOTES AND QUERIES.

By our Travel Correspondent.

Lodgings at Colwyn Bay (B. W. T.).—Thanks for information; always most useful when personally vouched for. The only thing to be advanced against the rooms (which sound ideal) is that, unfortunately, the terms are beyond the means of most of our correspondents, who, speaking generally, are nurses or hard-working doctors.

Holidays in September (Erin).—There is no charge for information in this column. I think Killarney is out of the question on your terms, and, even were you wealthy, I fear you would find only one week's stay there, with such a long journey at each end, by no means a rest. You might manage the lakes, though that, too, means a very long and expensive journey. The expense would be less than if you went to Ireland. Ambleside, for instance. Return third-class tourist ticket, £2. Hotels dear. The most reasonable "County Temperance Inn," Lowwood, a little way from Ambleside, along the lake shore. You would do better at a boarding house at your terms. Mrs. Knipe, Greenend Farm, Hawkeshead, would charge you from about 30s. At Troutbeck, on Windermere, Miss Castree Drummermire charges from 25s. Mrs. Robinson, Moss Cottage, Brookside, Windermere, from about 30s. At Ullswater, try "Wood's Temperance Hotel." Coaches and steamers are numerous in the district and not very dear. If you like the Isle of Man, try Mrs. Denny, 5 Falcon Cliff Terrace, Douglas. This is a house much liked. You must use my name. Terms, from about 30s., according to requirements. If you would like to go abroad and would not mind extreme quiet, I could find you something that would be considerably cheaper. Let me hear again if these ideas do not suit, but write at once on reading thi answer, or you will not get your information the following week.

Rules in Regard to Correspondence for this Section.—All questioners must use a pseudonym for publication, but the communication must also bear the writer's own name and address as well, which will be regarded as confidential. All such communications to be addressed "Travel Editor, 'Nursing Section of *The Hospital*,' 28 Southampton Street, Strand." No charge will be made for inserting and answering questions in the inquiry column, and all will be answered in rotation as space permits. If an answer by letter is required, a stamped and addressed envelope must be enclosed, together with 2s. 6d., which fee will be devoted to the objects of "The Hospital" Convalescent Fund Any inquiries reaching the office after Monday cannot be answered in "The Hospital" of the current week.

### Everybody's Opinion.

### A DELICATE QUESTION.

"Superintendent" writes:—Lately I have been much troubled by the nurses of my staff going for walks with gentlemen. The nurses are mostly tradesmen's daughters, and I do not consider it right that gentlemen should be so friendly with them. So far I have not objected to the nurses walking with men of their own station in life. Now I have learnt that some of them are going for country walks, and meeting, whenever they are out, married men. I do not see how I am to prevent these things unless I make a rule that nurses must not meet or walk about with any of the male sex. This is only a small place, and I know that any little thing a nurse may do is noticed at once and talked about, whereas in a large place no one would even notice more important actions. Both doctors and patients round here prefer the nurses wearing out-door uniforms, so I am obliged to have it worn. When I am able to let a nurse go for trips, theatres, or dances, I always let her wear private dress, so that she can feel free from remark. Matters have come to such a pass that I feel something must be done, as otherwise the Home will get a bad name. Will you give me your kind advice upon this matter, as it is one of great importance to me? I like the nurses to be happy and enjoy themselves, but I must put a stop to their being talked about as they now are. If I make a rule forbidding any such walking or meeting, and that if they break that rule they are liable to instant dismissal, would you consider that legal, and a right thing to do? If not, what course would you advise?

### THE NEW RULES AT ST. ANDREW'S HOUSE CLUB.

"Also a Member" writes:—As a member of St. Andrew's House Club, may I say that I think the remarks made by your correspondent regarding it distinctly unfair? The term "private hotel" means very little in reference to expense; in its cheaper days this club itself was called an "hotel," and there are many such to be found cheaper than St. Andrew's House. As the house and management remain the same as before, it is not clear why it should be less of a "home" on account of increasing prices. As to our disillusionment, we were allowed to believe, unofficially, that the club was only expected to pay its own expenses, which seems to be quite compatible with the explanation now given that the altered charges are to meet extra expenses. The servants, whose kitchen is on the fourth story, do not offer to enter the lift before or with the members, on whose behalf they use it, and for whose use it never was undertaken to be reserved; few would grudge this necessary boon, which is not unduly taken advantage of, and no one would expect the lift to be in use at other hours than those mentioned by your correspondent. The bathrooms leave nothing to be desired, the baths are clean, sanitary, and in good order; the enamel wears off from time to time, even now they have been re-enamelled by a harder process. These complaints, I think, will not be endorsed by the members, to whom they are more likely, rather, to give offence. The real grumblers of St. Andrew's House are few, and might be counted on the fingers of one hand, if not the thumb. We are all as sorry as your correspondent about the high prices, especially on behalf of those of us already belonging to the club who may not feel able to use it as freely as hitherto, also on account of other eligible nurses who would be able to appreciate this nice house if they were not excluded by the (to them) too high terms.

Mrs. Eleanor Ebbetts, late sister-superintendent of the Hampstead General Hospital writes: My attention has been called to a letter from a member of St. Andrew's House Club in The Hospital of August 27 concerning the management of that club. The tone of the letter must necessarily be deplored by members of the club. I have been a member of the club from the commencement and have stayed at the club several times for some weeks. I consider that there is no foundation for the complaints made about the management. The charges must be considered moderate by any impartial person, for the food is excellent in quality, very nicely served, and infinitely better in every way than at many hotels and *pensions*, and certainly very much cheaper. A member of the Reform and Travellers' Clubs (who dined with me at St. Andrew's Club a few weeks ago) told me that there was nothing better that he had at either of those clubs and that St. Andrew's was the best ladies' club he had seen. The additional charges referred to in the members' letter must be the small extra charge for bedrooms, a small weekly charge for the electric lift, and probably the notice that the boarding terms will be discontinued. I have had no communication with the proprietress over this matter; she does not know that I am writing to you, and I am not a member of the club committee. However, I have heard indirectly that the club did not pay its expenses last year. When I remember what I have seen —the abuse of the electric light and lift and the advantage taken of the very cheap boarding terms by some of the members, I can only express my surprise that the proprietress has hitherto been so long-suffering, and that she has not raised the charges long before this. Every effort has been made to ensure the comfort of the members at a minimum charge, and I think it due to the proprietress, who has taken so much trouble over it, to record the satisfaction felt by many members besides myself. In conclusion, allow me to add that if any member objects to the way the club is managed, she can appeal to the committee, or if that does not commend itself to her, the obvious and desirable course from every point of view is that she should leave a club that does not suit her. I note that The Hospital with its usual fair-minded and common-sense view holds the same opinion.

### Appointments.

Blackwall Branch Asylum.—The three following nurses have been appointed on the staff of the above institution. Miss E. M. Dowling, who was trained at the Wandsworth and Clapham Union Infirmary; Miss G. E. Peacock, trained at the Bethnal Green Workhouse Infirmary; and Miss C. G. Crease, trained at the Wandsworth and Clapham Union Infirmary.

Bolton Union Infirmary.—Miss E. N. Watson has been appointed charge nurse. She was trained at the Union Infirmary, Kingston-upon-Thames.

Borough Hospital, St. Helen's, Lancashire.—Miss I. Millar has been appointed sister. She was trained at the Fleming Hospital, Newcastle-on-Tyne, and has since acted as staff nurse at the City Hospital, Park Hill, Liverpool.

City Hospital, Bradford.—Miss Mary A. Elliott has been appointed head nurse. She was trained at the Kent and Canterbury Hospital. She has since been sister at the Johnstone Combination Hospital, and nurse-matron of the Saltwell Hall Hospital, Gateshead.

City Hospital, Walker Gate, Newcastle-on-Tyne.—Miss Harriet Deakin has been appointed night superintendent. She was trained at the New Infirmary, Birmingham; St. George's Private Nursing Home, Sheffield; and the General Infirmary, Leeds, where she obtained a first-class certificate and was appointed day sister.

Clapham Maternity Hospital.—Miss Frances E. Brown has been appointed sister. She was trained at Guy's Hospital, London. She has since been first assistant nurse at the Western Fever Hospital, London; sister at the Metropolitan Convalescent Institution, Walton-on-Thames; and nurse on the staff of Guy's Trained Nurses' Institution. She holds the L.O.S. certificate and is a registered midwife.

Milton Workhouse Infirmary.—Miss Ada B. Clarke has been appointed matron. She was trained at the Birmingham Infirmary, and has since been charge nurse at Great Yarmouth Isolation Hospital, home sister, night superintendent, and assistant matron at Stockport Union Infirmary and matron at Steyning Union Infirmary.

Sarah Acland Memorial District Nurses' Home, Oxford.—Miss Alice H. Ellrington has been appointed superintendent. She was trained at Salisbury Infirmary. She has since been Queen's nurse at Bolton; Queen's nurse and assistant superintendent of Queen's nurses at Windsor; superintendent at Shaw Street, Liverpool; and inspector of Queen's nurses, under the Jubilee Institute, for the Midland Counties. Miss Ellrington holds the L.O.S. certificate.

### Echoes from the Outside World.

### Movements of Royalty.

BOTH the King and Queen returned to London on Saturday. In connection with his Majesty's visit to Marienbad, Dr. Ott stated before his departure that, "while the King had come in very good health, the cure has had very beneficial effects, and the King leaves Marienbad in the most perfect health in every respect, and declares that he never felt better in his life." On the arrival of his Majesty at Charing Cross Station at half-past four, a very large crowd had assembled and gave his Majesty a hearty greeting. The King repeatedly bowed and saluted in response. On Monday his Majesty left town for Rufford Abbey, the Queen also leaving for Copenhagen. She was accompanied by the Princess Victoria, and the royal yacht with her Majesty and the Princess on board had to wait in Sheerness Harbour for the rough weather in the North Sea to subside.

### Betrothal of the German Crown Prince.

ON Sunday, at the Kaiserhof, Altena, the Kaiser announced the betrothal of his eldest son, the Crown Prince, to the Duchess Cecilia Augusta Marie of Mecklenburg-Schwerin. The announcement was received with great enthusiasm. The German Crown Prince, Frederick William, was born at Potsdam on May 6th, 1882, and the Duchess Cecilia at Schwerin on September 20th, 1886. She is described as blonde and pretty, and is an excellent linguist. Her brother is the reigning Grand Duke of Mecklenburg-Schwerin.

### The Battle of Liau-yang.

THE most important stage of the struggle between the Japanese and the Russians, so far, is the battle of Liau-yang, which raged for several days and ended with the Russians being compelled to effect a retreat. This, though not decisive, is, of course, a great victory for the Japanese, who have now taken possession of the place, with Marshal Oyama in command. It is stated that about half a million of men were engaged in the struggle, and the losses on both sides must have been enormous. The railway station was wrecked by shell-fire. According to General Sakharoff, the losses of the Russians in two days, August 31st and September 1st, were 7,000, and he estimates those of the Japanese at double or treble that number.

### A Royal Commission on Idiots.

THE King has approved the appointment of a Royal Commission to consider the existing methods of dealing with idiots and epileptics, and with imbecile, feeble-minded, or defective persons not certified under the lunacy laws; and, in view of the hardship or danger resulting to such persons and to the community from insufficient provision for their care, training, and control, to report as to the amendment in the law or other measures which should be adopted in the matter, due regard being had to the expense involved in any such proposals as to the best means of securing economy therein. The chairman of the Commission is the Marquis of Bath, and Mr. C. S. Loch and Mrs. Pinsent are among the members.

### New Governor-General of Canada.

THE appointment of Earl Grey as Governor-General of the Dominion of Canada, in succession to the Earl of Minto, is officially announced. Lord Grey, who is a brother-in-law of Lord Minto, was born in 1851. His father, General Charles Grey, was Equerry and Private Secretary to Queen Victoria, and previously Private Secretary to the Prince Consort. He himself was educated at Harrow, and Trinity College, Cambridge, where he had a distinguished record. As Mr. Albert Grey he represented South Northumberland in the Liberal interest from 1880 to 1885, and the Tyneside Division from 1885 to 1886. He subsequently joined the Liberal Unionists. In 1894 he succeeded to the title and family honours at the death of his eminent uncle. From 1896 to 1897 he was Administrator of Rhodesia. He is Chairman of the Public-house Trust Fund, and President of the Colonial Nursing Association. The Countess Grey is sister of Captain G. L. Holford, of Westonbirt, Gloucestershire, and is very popular in society.

### New Diplomatic Appointments.

SIR EDWIN HENRY EGERTON has been appointed Ambassador to Italy in succession to Sir Francis Bertie, who was lately presented to the Ambassadorship to France; and Sir J. Rennell Rodd has been appointed Minister Plenipotentiary at Stockholm in succession to Sir William Barrington. Sir Edwin Egerton is 63, and has been Ambassador at Madrid since last November. He was previously Secretary of Legation at Buenos Ayres and Athens, returning to the capital of Greece in 1892 as British Minister. He has also been Consul-General in Egypt, and has served at Constantinople and Paris. While he was at Athens he married a Russian lady, the daughter of Prince Nicholas Lebanoff Rostoffski. Sir Rennell Rodd is 46, and he has had 21 years' experience in the diplomatic service. He has held appointments at Berlin, Athens, Rome, and Paris. Since 1901 he has been Secretary of the Embassy at Rome. He is a poet of distinction, the biographer of the Empress Frederick, and has interested himself in the Anglo-American Nursing Home.

### More Alpine Accidents.

YET another serious disaster has occurred in the Alps, the victims being four Englishmen, who last week lost their lives in climbing on the Grand Paradis. The names of the unfortunate men are W. L. Clay, T. L. Winterbotham, Meryon, and the Rev. W. E. Wright, who are stated to have decided to make the ascent of the mountain without guides, against the strong representations of their friends. The Grand Paradise, which is on the Italian side of Mont Blanc, is 13,324 feet above sea-level, and is the highest of the Alpine mountains situated wholly in Italy. It is accessible either from Ponte, Courmayeur, or Cogne, but the four climbers were warned that the ice with which the ridge is incrusted was in a highly dangerous condition owing to the heavy snow which had recently fallen. On Friday evening last a carriage containing three passengers—Mr. John Fawcett, his daughter, and Mr. C. W. Parker—all of London, went over the side of the Schöllenen Road and rolled down a steep ravine. Mr. Fawcett was killed at once, and Mr. Parker, who had both his legs broken, died while he was being conveyed to Lucerne in an ambulance. Miss Fawcett and the driver sustained slight injuries.

### The Last of the Sea-trips.

THE sea-trips from London on the New Palace steamers will cease on Monday next and those who wish to take advantage of them must, therefore do so at once. The popularity of the service may be judged by the fact that during the summer months the *Royal Sovereign* and the *Koh-i-nor* have carried many thousands of passengers, the trips to Deal and Dover having been especially well patronised. No doubt this is due to the cleanliness of the steamers and the admirable catering.

### A Book and its Story.

NEW NOVEL BY THE AUTHOR, "THE OLD DOMINION," ETC.*

Miss Mary Johnstone's new novel, "Sir Mortimer," is a fascinating sixteenth-century romance in which the adventures of the gallant ships the *Mere Honour*, the *Cygnet*, the *Marigold*, and the *Star* are set forth in the admirable style characteristic of Miss Johnstone's writing. To Sir John Nevil was given the command of the little fleet whose ostensible destination was the Spanish Main, with an eye also to any adventures, encountered *en route*, which would bring fame and treasure in their train.

The story opens on the day before the vessels set sail. They are lying above Greenwich, and Sir Mortimer Fern stands, winecup in hand, addressing the assembled company in the parlour of the "Triple Tun Tavern." The *Mere Honour* was a vessel which shared the distinction of being one lent by Queen Elizabeth for the expedition, and of being the largest of those under Sir John's command. The tavern of the "Triple Tun" rang with acclamation, and waylaid the ears of them who went busily to and fro, and of them who lounged in the doorway or with folded arms played atlas to the tavern walls. "Who be the roisterers, William?" demanded a passing citizen of one of these supporters. The latter made no reply, but his neighbour, a boy in blue and silver, squatted upon a sunny bench, vouchsafed enlightenment. "Travellers to strange places," quoth he; "tall men . . . my master, Sir Mortimer Ferne, captain of the *Cygnet*, and his guests to dinner, Sir John Nevil, Admiral of our Fleet, with sundry of us captains and gentlemen-adventurers, and for a seasoning a handful of my master's poor friends, such as courtiers and great lords and poets." Sir Mortimer is described as "a man about thirty years, richly dressed and out of reason, good to look at." He addresses his guests in the characteristic diction of the period: "If we return not from our adventure, if the sea claims us, if strange islands know our resting-place, sunk for evermore in huge unkindly forests, if being mere pawns in a mighty game we are lost or changed, happy, however, in that the white hand of our Queen hath touched us, giving thereby consecration to our else unworthiness—if we find no gold, nor take one ship of Spain any city treasure stored—if we suffer a myriad sort of sorrows and at the last perish miserably ——" He paused, and raised the winecup: "I drink to those who follow after!" I drink to those who fail—pebbles cast into the water whose ring still wideneth, and reaches, God knows what unquestable shore, where loss may yet be counted gain! I drink to Fortune her minions, to Francis Drake and John Hawkins, Martin Frobisher; to all adventurers in the far-off seas! I drink to Merry England and to the day when every son shall bring her tribute to England, like Aphrodite, new-risen from the seas' main!"

Between Sir Mortimer's page, the boy in blue and silver who stood outside the door of the Triple Inn while the parting speech was being made, there was a mutual feeling of affection on the one hand and devotion on the other. His master adored the fair sister of a young officer, sailing in the *Cygnet*, who was a maid-of-honour to the Queen. Before starting he had sought an opportunity of meeting her alone. Aided by Henry Sedley's directions he found the fair Damaris. In the following charmingly quaint description she was made known to him. "Beneath a great oak tree, where light and shadow made a chequered round, Mistress Damaris sadly sat upon the earth in a gown of rose-coloured silk. Across her knee, under her clasped hands, lay a light racquet, for she had strayed this way from battle-door and shuttlecock, and the sprightly company of maids-of-honour and gentlemen-pensioners engaged thereat. She was a fair lady, of a clear pallor, with a red mouth very subtlely charming, and dark eyes between level brows. Her eyes had depths on depths: to one they were merely large brown orbs; another might find in them worlds above worlds; a third, going deeper, might, Acteon-like, surprise a bare soul. A curiously wrought net of gold caught her dark hair in its meshes; pearls were in her ears and around the white column of her throat, rising between the ruff's gossamer walls." Sir Mortimer had worshipped in secret for some time, and under the classic name of Dione had approached in verse the Lady Damaris. To her young brother the knight was a hero whom he held in almost poetic admiration. This sea captain, this man of war who was yet a courtier and a scholar, "the violet knight who was yet to lead him up to heights which long ago the knight himself had scaled." The chapter in which the meeting of Sir Mortimer and his lady is described, and which ends in a perfect piece of Elizabethan love-dialogue, with its quaint, tender metaphor, is a sunny interlude between the more strenuous scenes of fighting and peril. "I must go forth to-morrow," he said, "to seek, to find, to win, to lose—God He knoweth what! I would go as your knight avowed, your favour in my helm, your kiss like holy water on my brow. See, I kneel to you for some charm, some sign, to make my voyage good. Very slowly the rose-clad maid of honour let her gaze fall from the evening skies to the man before her; as slowly she unclasped her hands so tightly locked behind her upraised head. . . . She bowed herself before him; a knot of rosy velvet, loosened from her dress, fell upon the turf beside his knee. Ferne caught up the ribbon, pressed it to his lips, thrust it in the breast of his doublet. Rising, he took her in his arms and they kissed. "My lady and my only dear," he cried. "Oh, Love is as the Sun! So the sunshine bide, let come what will come!" "I rest in the sunshine," she said. "Oh, Love is bliss . . . but anguish too! I see the white sails of your ships." She shuddered in his arms. "All that go return not. Ah, tell me that you will come back to me!" "That I will do," he answered, "an I am a living man. If I die, I shall but wait for thee. I see no parting of our ways."

A vivid sketch of Queen Elizabeth as she makes her royal progress must close the notice of this most interesting and dramatic picture of her remarkable reign.

"Burleigh on one hand, Leicester on the other, encompassed and followed by the greatest names and the fairest faces of England herself erect, ablaze with jewels, conscious of her power, and at all times ready to wield it, came the daughter of Henry the Eighth. A noble presence, moving in the full lustre of sovereignty, a princess who, despite all womanish faults, bore a wise king unto her people, a maiden ruler to whom in that aftermath of chivalry men gave a personal regard, rose-coloured and fanciful; the woman not above coquetry, vanity, and double dealing; the monarch whose hand was heavy upon the council board, whose will perverted law, and whose prime wish was the welfare of her people." Later Sir Mortimer returns from Spain, a self-indicted traitor. His love, although broken-hearted at the rumours of his treachery to his country, cannot believe him guilty. He receives the sentence of banishment from the Queen to his own estate, where he is kept prisoner for four years. Then the truth comes out; the result is not for us to give away, and so forestall the *dénouement* of this charming romance.

* Sir Mortimer. By Mary Johnstone. (Constable. 6s.)

### Notes and Queries.

**FOR REGULATIONS SEE PAGE 143.**

*Medical Etiquette.*

(177) 1. Would it be permissible for a nurse to perform tracheotomy in case of urgency to save life when it is impossible to secure medical aid in time? 2. Would it be etiquette for a patient at a health resort to call in his own medical attendant when there was a resident doctor?—*Aubrey.*

1. In very exceptional instances a nurse would be justified in performing laryngotomy (not tracheotomy); for example, when a patient was in the last stage of suffocation, and the obstruction could not be removed through the mouth and medical aid was remote. But the operation is not so easy as it might seem to one who has never performed it, and it would offer but small hope of success unless the nurse was thoroughly familiar with every step of the operation from frequently seeing it performed. 2. There is no rule of etiquette which prevents a patient from consulting his own medical attendant.

Nurses F. and N. would be grateful if any reader of The Hospital would kindly give their opinion as to whether or not it is etiquette for a doctor to cold-sponge a patient when there are two competent and certificated nurses present?

A nurse is subordinate to the medical attendant, and must act on his instructions.

*Address.*

(178) Kindly give me the addresses of specialists on bowel complaints in London, Manchester, or Liverpool?—*Nurse S.*

We cannot give the addresses of specialists. Consult your medical man, and allow him to recommend you a specialist.

(1) May I trouble you for the address of the Royal British Nurses' Association? To whom should I apply for membership? (2) What London hospital would be most likely to accept a probationer aged 34?—*Doubtful.*

(1) Write to the Secretary, 10 Orchard Street, W. (2) See "The Nursing Profession: How and Where to Train."

*Abroad.*

(179) Will you kindly tell me if there is any pamphlet giving collective information with regard to homes for invalids on the French and Italian Rivieras at a cost of 20s. or 25s. a week?—*B. K. F.*

We know of no pamphlet giving the information required, but there is a short list in the "Englishwoman's Year Book."

Will you kindly tell me the addresses of homes in Rome where nurses are supplied for private work?—*Scot.*

The Anglo-American Nursing Home, 265 Via Nomentana; and St. Paul's Home for Nurses, 25 Via Vincensa, Rome.

Will you kindly tell me where I can obtain information as to nursing prospects in Canada? I want to find out all particulars before emigrating.—*P.M.E.W.*

Write to the Lady Superintendent, the Victorian Order of Nurses for Canada, 4578 Somerset Street, Ottawa.

*Sanatoria.*

(180) Will you kindly tell me if there are any sanatoria for the open-air treatment of consumption in the neighbourhood of Carlisle, where a lady suffering from phthisis in an advanced stage could be received for a small sum?—*A. E. R.*

See the list of Sanatoria, published by the National Association for the Prevention of Tuberculosis, obtainable from The Scientific Press, price 7d. with postage.

Will you kindly tell me where I can get a list of institutions for the open-air treatment for consumption, where poor patients are received free of charge?—*Miss Y.*

See reply to *A. E. R.*

**Important Nursing Text-books.**

"The Nursing Profession: How and Where to Train." 2s. net; 2s. 4d. post free.

"The Nurses' Dictionary." By Honnor Morten. 2s. post free.

"Nursing: a Complete Text-book of Medical, Surgical, and Monthly Nursing." By Percy Lewis, M.D. 3s. 6d., post free.

"Elementary Physiology for Nurses." By C. F. Marshall, M.D. 2s. post free.

"A Complete Handbook of Midwifery for Nurses." By J. K. Watson, M.D. 6s. net; 6s. 4d. post free.

The above works are obtainable of all booksellers, or direct from The Scientific Press, Limited, 28 & 29 Southampton Street, Strand, London, W.C.

### For Reading to the Sick.

CONVALESCENCE.

Teach me to live, no idler let me be,
But in Thy service hand and heart employ,
Prepared to do Thy bidding cheerfully—
Be this my highest and my holiest joy.

Teach me to live—my daily cross to bear,
Nor murmur though I bend beneath its load,
Only be with me; let me feel Thee near,
Thy smile sheds gladness on the darkest road.

Teach me to live and find my life in Thee,
Looking from earth and earthly things away.
Let me not falter, but untiringly
Press on, and gain new strength and power each day.

*E. E. Burman.*

"She arose and ministered unto them."—St. Matt. viii. 15.

The figure of our beloved Lord is so continually presented to us in the Gospels as that of "a Man of sorrows and acquainted with grief," that we scarcely ever think of Him as rejoicing in the pure joys of humanity, but only as weeping with its sorrows and sharing in its sufferings. Yet, let us wander for a few moments in thought along the shores of the Galilean lake; let us enter the house of Peter, the fisherman, and see how bright with joy are the faces of its inmates; see how the anxiety of supplication is giving place to the rejoicing of a fervent gratitude; for the Divine Master has restored to them the beloved object of their intercessions and raised her with His stong and tender hand from the bed of suffering.

But while we may gather from this incident our Lord's sympathy with holy joy, let us not pass by the teaching which lies in those few short and touching words, "And she arose and ministered unto them." Here let us learn the lesson of a devout thankfulness as it is expressed, not in words, but in deeds.

Arise thou who hast been restored to life by the touch of Jesus, forget thyself and thy sufferings, whatever they may have been, or remember them only that through their experience thou mayest comfort others; go forth on thy ministry according as God may call thee; in the home of gladness or in the world of suffering and sin. He will give thee thy work, if only thou wilt arise; and in that day when sorrow and sighing shall flee away, the voice of Jesus shall fall upon thy wondering ear in gracious accents from the heavenly shore, saying, "Well done, good and faithful servant, enter thou into the joy of thy Lord. Inasmuch as thou hast done it unto one of the least of these My brethren, thou hast done it unto Me."—*M. E. Townsend.*

I would have gone; God bade me stay;
I would have worked; God bade me rest.
He broke my will from day to day,
He read my yearnings unexprest
And said them nay.

*C. Rossetti.*

